# Supplementary material for: Spontaneous and stimulus-driven arousal produce distinct acetylcholine dynamics across sensory and prefrontal cortex
Source: bioRxiv. 2026 Jun 3:2026.06.02.729441. Preprint. [Version 1] doi: 10.64898/2026.06.02.729441 (PMC13251989; doi:10.64898/2026.06.02.729441)
Supplement: Supplement 1 [file media-1.pdf]

Supplemental information

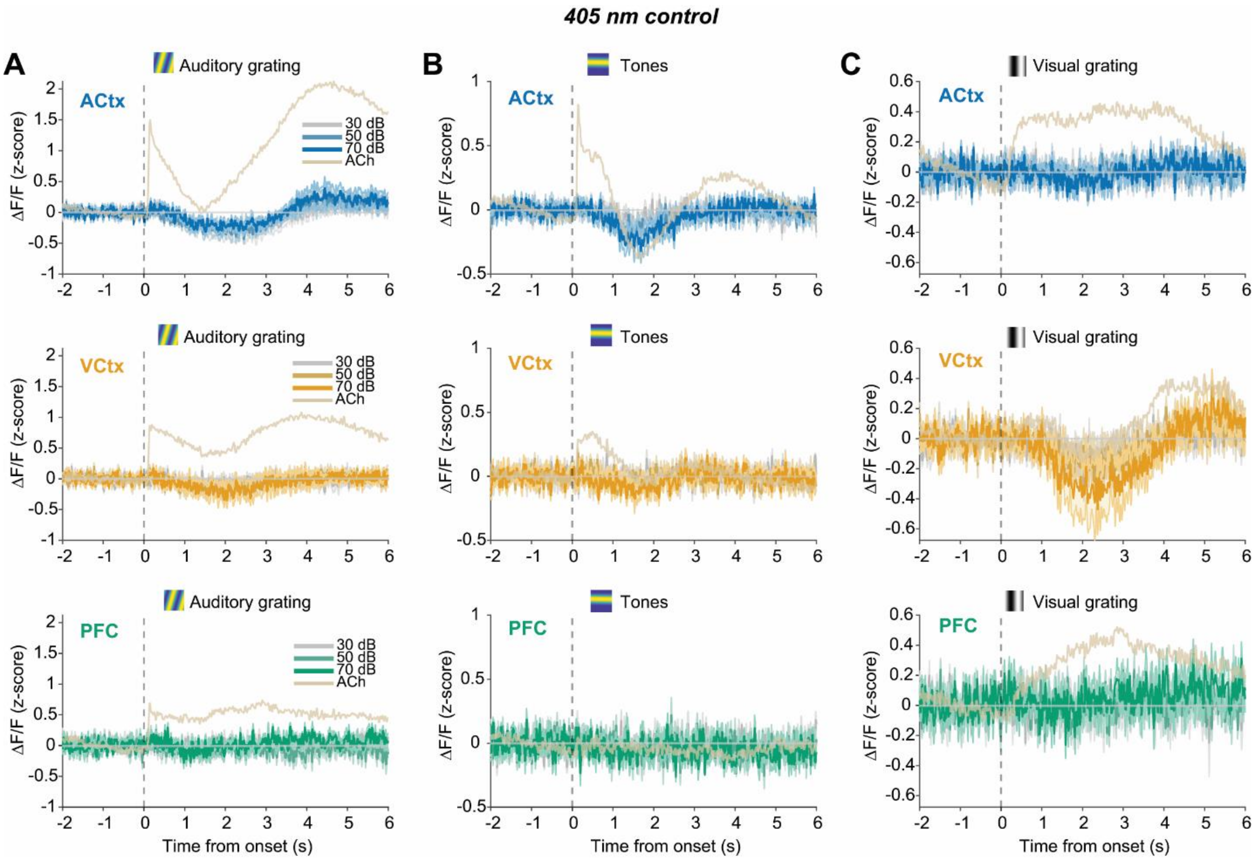

FIGURE S1. (**A**) Auditory grating-evoked DF/F traces (z-scored) at different intensities, across ACtx, VCtx, and PFC (top to bottom), in the 405 nm isosbestic control condition. Beige trace in each plot shows the corresponding ACh (465 nm) response in each cortical region, pooled across intensities, for comparison. (**B-C**) Same as **A**, but for tones (**B**) and visual gratings (**C**).

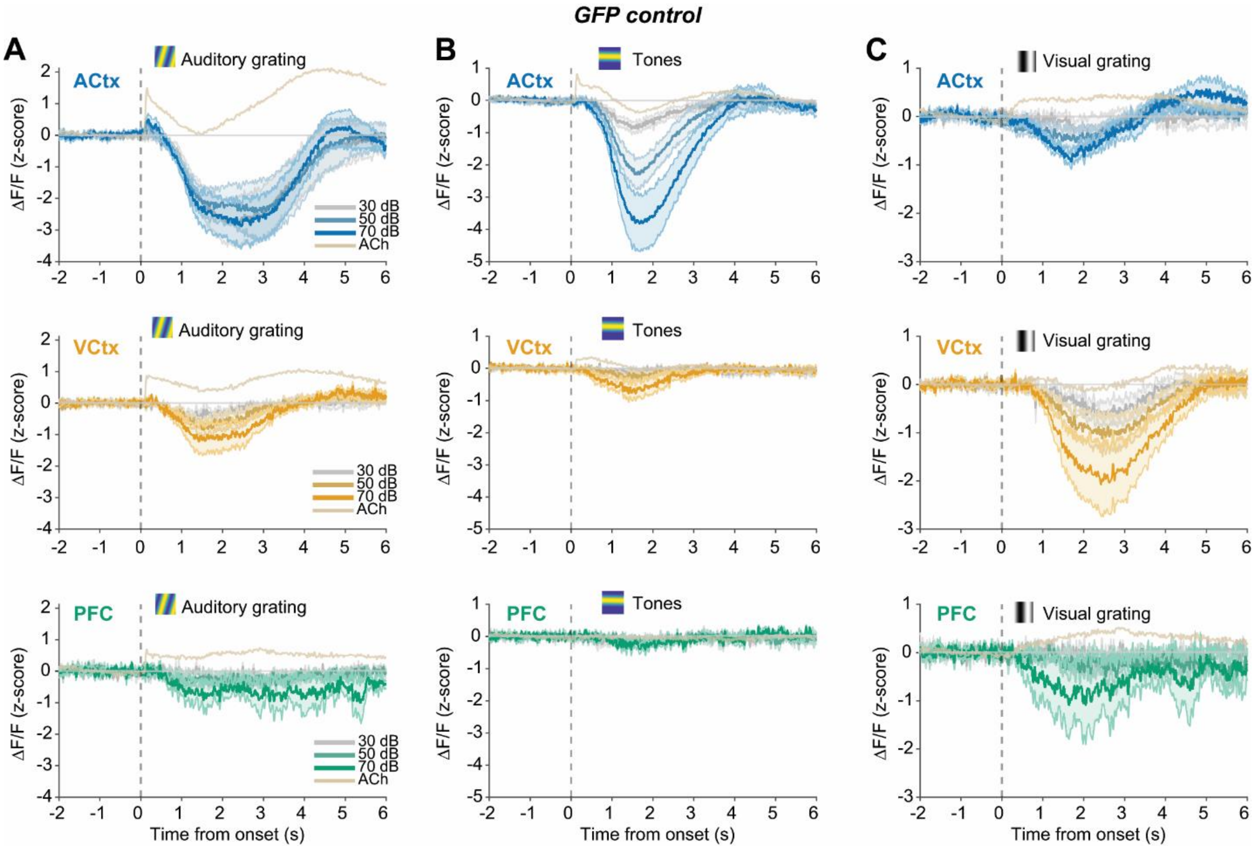

FIGURE S2. (**A**) Auditory grating-evoked DF/F traces (z-scored) at different intensities, across ACtx, VCtx, and PFC (top to bottom), in mice expressing the fluorophore GFP in cortex, rather than the ACh indicator. Beige trace in each plot shows the corresponding ACh (465 nm) response in each cortical region, pooled across intensities, for comparison. (**B-C**) Same as **A**, but for tones (**B**) and visual gratings (**C**).

Signal	Stimulus	Region	Intensity	n_mice	Mean_AUC	SEM_AUC	p_raw_Wilcoxon
ACh	Aud. Ripple	AC	30 dB	11	0.553	0.1994	0.002
ACh	Aud. Ripple	AC	50 dB	11	0.5739	0.2357	0.002
ACh	Aud. Ripple	AC	70 dB	11	0.9538	0.2711	0.002
ACh	Aud. Ripple	VC	30 dB	7	0.4931	0.1293	0.0156
ACh	Aud. Ripple	VC	50 dB	7	0.6589	0.1374	0.0156
ACh	Aud. Ripple	VC	70 dB	7	0.7577	0.1603	0.0156
ACh	Aud. Ripple	PFC	30 dB	5	0.0747	0.1104	1
ACh	Aud. Ripple	PFC	50 dB	5	0.3184	0.1725	0.1875
ACh	Aud. Ripple	PFC	70 dB	5	0.8759	0.2061	0.0625
ACh	Tones	AC	30 dB	11	0.2507	0.0862	0.0098
ACh	Tones	AC	50 dB	11	0.3174	0.1159	0.0098
ACh	Tones	AC	70 dB	11	0.4763	0.1642	0.0068
ACh	Tones	VC	30 dB	7	0.0214	0.0307	0.8125
ACh	Tones	VC	50 dB	7	0.2821	0.0872	0.0156
ACh	Tones	VC	70 dB	7	0.4038	0.111	0.0156
ACh	Tones	PFC	30 dB	5	-0.0581	0.05	0.3125
ACh	Tones	PFC	50 dB	5	-0.063	0.0721	0.625
ACh	Tones	PFC	70 dB	5	0.039	0.0359	0.4375
ACh	Vis. Grating	AC	0.11	11	0.0638	0.0571	0.5771
ACh	Vis. Grating	AC	0.33	11	0.01	0.0548	0.5771
ACh	Vis. Grating	AC	1	11	0.6029	0.1637	0.001
ACh	Vis. Grating	VC	0.11	7	-0.101	0.0699	0.1562
ACh	Vis. Grating	VC	0.33	7	0.0365	0.0659	0.6875
ACh	Vis. Grating	VC	1	7	0.2271	0.0588	0.0312
ACh	Vis. Grating	PFC	0.11	5	-0.1016	0.0683	0.3125
ACh	Vis. Grating	PFC	0.33	5	-0.028	0.0216	0.4375
ACh	Vis. Grating	PFC	1	5	0.3771	0.1168	0.0625

GFP	Aud. Ripple	AC	30 dB	6	-0.2095	0.1164	0.1562
GFP	Aud. Ripple	AC	50 dB	6	-0.2314	0.1007	0.0938
GFP	Aud. Ripple	AC	70 dB	6	-0.1262	0.1251	0.5625
GFP	Aud. Ripple	VC	30 dB	6	-0.1292	0.0395	0.0312
GFP	Aud. Ripple	VC	50 dB	6	-0.1416	0.062	0.1562
GFP	Aud. Ripple	VC	70 dB	6	-0.1672	0.0564	0.0312
GFP	Aud. Ripple	PFC	30 dB	3	-0.0369	0.0227	0.25
GFP	Aud. Ripple	PFC	50 dB	3	-0.1007	0.0642	0.25
GFP	Aud. Ripple	PFC	70 dB	3	-0.1695	0.0798	0.25
GFP	Tones	AC	30 dB	6	-0.0976	0.0456	0.1562
GFP	Tones	AC	50 dB	6	-0.2162	0.1044	0.1562
GFP	Tones	AC	70 dB	6	-0.4568	0.1132	0.0312
GFP	Tones	VC	30 dB	6	-0.0379	0.0227	0.1562
GFP	Tones	VC	50 dB	6	-0.0644	0.0427	0.2188
GFP	Tones	VC	70 dB	6	-0.2127	0.104	0.0625
GFP	Tones	PFC	30 dB	3	-0.0982	0.0873	0.5
GFP	Tones	PFC	50 dB	3	-0.0095	0.0282	0.75
GFP	Tones	PFC	70 dB	3	-0.0194	0.0174	0.5
GFP	Vis. Grating	AC	0.11	6	-0.0315	0.0841	1
GFP	Vis. Grating	AC	0.33	6	-0.244	0.0987	0.0312
GFP	Vis. Grating	AC	1	6	-0.1551	0.0692	0.1562
GFP	Vis. Grating	VC	0.11	6	-0.0946	0.0552	0.3125
GFP	Vis. Grating	VC	0.33	6	-0.0321	0.0706	0.4375
GFP	Vis. Grating	VC	1	6	-0.0647	0.0305	0.1562
GFP	Vis. Grating	PFC	0.11	3	0.0253	0.0356	0.5
GFP	Vis. Grating	PFC	0.33	3	-0.0803	0.0404	0.25
GFP	Vis. Grating	PFC	1	3	-0.2199	0.2227	0.75

405nm	Aud. Ripple	AC	30 dB	10	-0.0084	0.0198	0.9219
405nm	Aud. Ripple	AC	50 dB	10	-0.0517	0.0304	0.1309
405nm	Aud. Ripple	AC	70 dB	10	0.0054	0.0399	0.4922
405nm	Aud. Ripple	VC	30 dB	7	0.0003	0.0072	0.9375
405nm	Aud. Ripple	VC	50 dB	7	-0.0473	0.0159	0.0312
405nm	Aud. Ripple	VC	70 dB	7	-0.0231	0.0284	0.8125
405nm	Aud. Ripple	PFC	30 dB	4	-0.0059	0.0055	0.625
405nm	Aud. Ripple	PFC	50 dB	4	-0.022	0.0112	0.125
405nm	Aud. Ripple	PFC	70 dB	4	0.0301	0.0162	0.125
405nm	Tones	AC	30 dB	10	0.0072	0.0115	0.4316
405nm	Tones	AC	50 dB	10	-0.0255	0.0246	0.625
405nm	Tones	AC	70 dB	10	-0.0207	0.0229	0.625
405nm	Tones	VC	30 dB	7	0.0049	0.0066	0.4688
405nm	Tones	VC	50 dB	7	0.02	0.0128	0.1562
405nm	Tones	VC	70 dB	7	-0.0301	0.0213	0.1562
405nm	Tones	PFC	30 dB	4	0.0021	0.0251	0.875
405nm	Tones	PFC	50 dB	4	-0.0104	0.0266	0.875
405nm	Tones	PFC	70 dB	4	-0.0256	0.0081	0.125
405nm	Vis. Grating	AC	0.11	10	0.022	0.0276	0.7695
405nm	Vis. Grating	AC	0.33	10	-0.0009	0.0182	1
405nm	Vis. Grating	AC	1	10	-0.0024	0.0137	0.6953
405nm	Vis. Grating	VC	0.11	7	-0.0198	0.0183	0.4688
405nm	Vis. Grating	VC	0.33	7	-0.0107	0.0219	0.375
405nm	Vis. Grating	VC	1	7	-0.0105	0.0159	0.6875
405nm	Vis. Grating	PFC	0.11	4	0.0057	0.0114	0.875
405nm	Vis. Grating	PFC	0.33	4	0.0311	0.0272	0.375
405nm	Vis. Grating	PFC	1	4	0.0256	0.0173	0.25

TABLE S1. Results from one-sided *t*-test comparing the distribution of mean AUC values (from 0-1 s post stimulus onset) of ACh, isosbestic control (405nm), or GFP z-scored DF/F in each cortical region, for each stimulus type.
